# Re-intervention rate in endovascular vs open surgical repair for abdominal aortic aneurysms

**DOI:** 10.1016/j.amsu.2021.102703

**Published:** 2021-08-10

**Authors:** Ahmed Abdel Rahim, Rashid Ibrahim, Lu Yao, Wei Shen cheok, Mohammed Ismail

**Affiliations:** a University Hospitals Plymouth NHS Trust, United Kingdom; b Aswan University Hospitals, Egypt

**Keywords:** Infrarenal abdominal aortic aneurysm (AAA), Endovascular aneurysm repair (EVAR), Open surgical repair (OSR), Re-intervention rates

## Abstract

A best evidence topic has been constructed using a described protocol. The three-part question addressed was: In patients with Infrarenal abdominal aortic aneurysm (AAA), Does endovascular abdominal aortic repair (EVAR), AS compared to open surgical repair (OSR), has lower re-intervention rates? The outcomes assessed were the re-interventional rates in both techniques. The best evidence showed that the OSR has lower statistically significant difference rates in re-intervention rates than the EVAR.

## Highlights

• Endovascular abdominal aortic aneurysm repair is inferior to the open surgical repair regarding the re-intervention rates.
• Long term outcomes of the open surgical repair are better than the Endovascular repair.
• Secondary procedures are more in endovascular repair than the open surgical repair of abdominal aortic aneurysm.

## 1 Introduction

This BET was designed using a framework outlined by the International Journal of Surgery [1]. This format was used because a preliminary literature search suggested that the available evidence is insufficient to perform a meaningful meta-analysis. A BET provides evidence-based answers to common clinical questions, using a systematic approach of reviewing the literature. (see Table 1)

### 1.1 Clinical scenario

While consenting a 50-year-old man with AAA for EVAR repair, one of the junior doctors asked; which modality of AAA repair has lower re-intervention rates; EVAR or Open repair?

Three Parts Question:

• [In patients with AAA,]
• [Which modality of treatment has lower re-intervention rates];
• [EVAR or OSR]?

### 1.2 Search strategy

1. Embase 1974 to June 2021 using the OVID interface:
[AAA OR Abdominal Aortic Aneurysm]AND [Open repair OR open surgical repair OR OSR] AND [EVAR OR Endovascular Repair] AND [re-intervention]
2. Medline using the PubMed interface:
[AAA OR Abdominal Aortic Aneurysm]AND [Open repair OR open surgical repair OR OSR] AND [EVAR OR Endovascular Repair] AND [re-intervention]

The results were limited to English articles and human studies.

• Inclusion criteria: all original articles that review the re-intervention rate among patients with AAA who underwent open surgical repair vs. Endovascular Repair.
• Exclusion criteria: case reports, systematic reviews, letters to the editor, conference abstracts.

### 1.3 Search outcome

A total of 261 papers were found using both search engines. We excluded Two hundred twenty-three essays because they were irrelevant based on the titles and or the abstracts. Thirty-eight full-text articles were screened and assessed for eligibility. From these, we identified six papers to provide the best evidence to answer the question.

## 2 Result: Table 1 search results

**Table 1:** Summary of search results.

Author/date of publication/journal/country	Study type and level of evidence	Patient group	Outcomes follow up		Key results	Additional comments
Lederle F A et al., 2019, N Eng J Med, USA [2]. OVER	Randomised control trial- Level 1b	Total of 881 patients with AAA * Group 1 EVAR: 444 *Group 2 OSR: 437 *Follow-up, 14.2 years. *Median was 9.4 years	*End point is re-interventions. *Other outcomes: all cause or aneurysm related mortality and Overall survival rate		*Group 1 EVAR: 26.7% (117) patients. *Group 2 OSR: 19.8% (85) patients. *95% confidence interval = 2.0–17.5. *P value = 0.04 *Statistically significant	*Long term *Multi Center *Specific skills and device training for the investigators.
Van Schaik et al., 2017, JVS, Netherland [3] Dream	Randomised controlled trial -level 1b	*Total of 351 patients with AAA *Group (1)OSR: 178 *Group(2) EVAR: 173 *Follow up was 12 years. * Median was 10.2 years.	*End point is re-interventions *Other outcomes: all cause or aneurysm related mortality and Overall survival rate		Group 1 OSR: 21.1% Group 2 EVAR: 37.8% * (95% confidence interval, 5.8–27.6) *P value = 0.01). *Statistically significant	* Long term follow up *Multi center *Lack of Blinding. *Old devices used
Rajesh Patel et al., 2016, Lancet, UK [4]. EVAR-1	Randomised control trial- Level 1b	*Total of 1252 patients with AAA *Group 1 OSR: 626 *Group 2 EVAR: 626 *Follow up was 15·8 years. *Median was 12.4 years.	*End point is re-interventions. *Other outcomes: all cause or aneurysm related mortality and Overall survival rate		*Group 1 OSR: 12% (74) Patients. *Group 2 EVAR: 26% (164) Patients. *(95% confidence interval,1.82–3.21) *P value < 0.0001 *Statistically significant	* Long term *Multi Centre *Old devices used * Imaging was of low quality *Follow up changed from CT to Ultrasound .
Huang et al., 2015, JVS, USA [5].	Retrospective Cohort -Level 2a	*Total of 1116 patients with AAA *Group 1 OSR: 558 *Group 2 EVAR: 558 *Follow-up, 10 years; *Median was 7.6 years.	*End point is re-interventions. *Other outcomes: all cause or aneurysm related mortality and Overall survival rate		Group 1 OSR: 8.06% (45) Patients Group 2 EVAR: 21.8% (122) Patients; * 95% confidence interval = 1.92–3.51; *P value < 0.001) *Statistically significant	*Large sample size *Retrospective *OSR group are less rigorously followed up.
Chang et al., 2015, JAMA Surg, USA [6].	Retrospective Cohort -Level 2a	*Total of 23670 patients with AAA. * Group 1 EVAR: 12239 (51.7%) Group 2 OSR: 11431 (48.3%) *Follow up is 9 years *Median was 3.3 years.	*End point is re-interventions. *Other outcomes: all cause or aneurysm related mortality and Overall survival rate		5 years Outcomes: *Group 1 EVAR: 169 patients (6.59%). *Group 2 OSR: 806 patients (1.48%) * 95% CI = 0.22–0.32 *P value < 0.001 *Statistically significant	*Large sample size *Multi center
Majd et al., 2017, Ann Vasc surg, Germany [7].	Retrospective Cohort -Level 2a	*Total of 177 patients with AAA. *Group 1 EVAR: 131 (74%). *Group 2 OSR: 46 (26%). *Follow up is 7 years *Median was 5 years for the OSR group and 4.5 years for the EVAR group.	*End point is re-interventions. *Other outcomes: Overall survival rate	5 years Outcomes: *Group 1 EVAR: 23 patients (17.6%). *Group 2 OSR: 4 patients (8.7%) *P-Value = 0.109 *Statistically insignificant		*Retrospective *Single center *Small sample size * selection bias

## 3 Discussion

Treatment of AAA has changed remarkably in the last decade. EVAR is now increasingly used to treat AAA, especially in high-risk and elderly patients [8]. This is because of improved perioperative outcomes and the less invasive repair than the open surgical repair. Although the overall survival rates of both interventions are equivalent, there are still a lot of question marks about the re-intervention rates and long-term outcomes [9].

The re-intervention rates are also called secondary procedures and are defined as any endovascular or surgical procedure done after the first intervention. This procedure may be directly or indirectly related to the First aneurysm repair [3].

In this article, we have reviewed the best studies that compared the two AAA repair modalities to evaluate which techniques have lower re-intervention rates.

Only one study in our review showed no statistically significant difference between EVAR and OSR in re-intervention rates; this study was conducted by Majid et al. [7]. This study is retrospective, single-center with a small sample size in addition to possible selection bias as OSR was reserved for more fit patients and EVAR for patients with high surgical risk and more suitable anatomy.

In contrast, there are another five trials; three of them were Randomized controlled trials which were conducted by Lederle FA et al. [2], Van Schaik et al. [3], and Rajesh Patel et al. [4], and another two retrospective cohort trials conducted by Huang et al. [5] and Chang et al. [6] show statistically significant lower re-intervention rates among patients with OSR in comparison to EVAR. All of these studies included large sample size and long periods of follow-up. However it is worth mentioning that bias must be considered in Van Schaik et al. and Rajesh Patel et al. because of using old devices.

## Clinical Bottom Line

According to the above articles, the best evidence shows a statistically significant lower re-intervention rate among patients with open surgical repair of abdominal aortic aneurysm in comparison to endovascular repair.

## Ethical Approval

Not required

## Source of Funding

None

## Author Contribution

AA: Conducted the literature search and wrote the paper. RI: Assisted in the literature search and Writing of paper. LY: Editing of writing. WSC: Assisted in writing of paper. MI: Assisted in the literature search and writing of paper.
